# A systematic review and meta-analysis of the diagnostic accuracy of the neutrophil-to-lymphocyte ratio and the platelet-to-lymphocyte ratio in systemic lupus erythematosus

**DOI:** 10.1007/s10238-024-01438-5

**Published:** 2024-07-25

**Authors:** Angelo Zinellu, Panagiotis Paliogiannis, Arduino A. Mangoni

**Affiliations:** 1 Department of Biomedical Sciences, University of Sassari, Sassari, Italy; 2 Department of Medicine, Surgery, and Pharmacy, University of Sassari, Sassari, Italy; 3 Anatomic Pathology and Histology Unit, Sassari University Hospital (AOU), Sassari, Italy; 4 Discipline of Clinical Pharmacology, College of Medicine and Public Health, Flinders University, Adelaide, Australia; 5 Department of Clinical Pharmacology, Flinders Medical Centre, Southern Adelaide Local Health Network, College of Medicine and Public Health, Flinders University, Bedford Park, Adelaide, SA 5042 Australia

**Keywords:** Systemic lupus erythematosus, Neutrophil-to-lymphocyte ratio, Platelet-to-lymphocyte ratio, Diagnostic accuracy, Severe SLE, Lupus nephritis, Infections

## Abstract

The wide range of clinical and serological manifestations in systemic lupus erythematosus (SLE) and the lack of accepted diagnostic criteria warrant the identification of novel, more accurate biomarkers. Hematological indices derived from full blood cell counts, particularly the neutrophil-to-lymphocyte ratio (NLR) and the platelet-to-lymphocyte ratio (PLR), have shown promise in SLE; however, a critical appraisal of their diagnostic accuracy is lacking. We sought to address this issue by conducting a systematic review and meta-analysis of the diagnostic accuracy of the NLR and PLR in SLE. The electronic databases PubMed, Scopus, and Web of Science were systematically searched from inception to 15 March 2024 for studies reporting the sensitivity and specificity of the NLR and PLR, obtained by receiver operating characteristic (ROC) curve analysis, for the presence of SLE, disease severity, organ involvement (lupus nephritis, pericarditis, and pleural disease), and complications (infections). The risk of bias was assessed using the JBI Critical Appraisal Checklist (PROSPERO registration number: CRD42024531446). The NLR exhibited good accuracy for the diagnosis of SLE (eight studies; area under the curve, AUC = 0.81, 95% CI 0.78–0.85) and lupus nephritis (nine studies; AUC = 0.81, 95% CI 0.77–0.84), but not for severe disease (nine studies; AUC = 0.69, 95% CI 0.65–0.73) or infections (six studies; AUC = 0.73, 95% CI 0.69–0.77). The PLR exhibited good accuracy for the diagnosis of severe disease (six studies; AUC = 0.85, 95% CI 0.81–0.87). There were an insufficient number of studies to assess the accuracy of the PLR for the diagnosis of SLE, lupus nephritis, or infections. No study investigated the NLR and PLR in SLE patients with pericarditis or pleural disease. Therefore, the NLR and the PLR have a relatively high diagnostic accuracy for the presence of SLE and lupus nephritis (NLR) and severe disease (PLR). Further studies are warranted to determine whether the NLR and PLR, in combination with clinical evaluation and other serological biomarkers, can enhance the diagnosis and management of SLE.

**Supplementary Information:**

The online version contains supplementary material available at 10.1007/s10238-024-01438-5.

## Introduction

Systemic lupus erythematosus (SLE) is a chronic autoimmune condition that can affect a wide range of organs and systems with different degrees of severity [1]. Although the production of anti-nuclear antibodies is a common feature of SLE, they have a relatively low specificity as they are also detected in other autoimmune conditions, cancer, infections, and healthy subjects [2, 3]. Furthermore, the heterogeneity of clinical manifestations and the lack of tests that are characteristic of the disease poses significant diagnostic challenges [4]. Such challenges may prevent early diagnosis and delay the commencement of effective therapies [5]. In absence of clear diagnostic criteria, several SLE classification criteria developed to categorize patients allowing their inclusion in clinical trials are used instead by clinicians. Such criteria include the 2019 EULAR/ACR criteria [6], the 2012 SLICC criteria [7], and the 1997 ACR criteria [8]. In validation cohorts, the 2019 EULAR/ACR criteria have shown a sensitivity of 96.1% and specificity of 93.4%, compared to 82.8% sensitivity and 93.4% specificity of the 1997 ACR criteria and 96.7% sensitivity and 83.7% specificity of the 2012 SLICC criteria [6]. However, the reported sensitivity and specificity of these criteria assumes the clinical assessment by experienced clinicians and a sufficient number of clinical features, which may not be present in patients in the early stages of the disease [9].

Several potential candidate biomarkers of SLE have been identified over the last 15 years, e.g., cell-bound complement activation products and anti-nucleosome antibodies. Other biomarkers have been proposed to monitor disease activity, e.g., anti-complement component 1q antibodies, interferon-alpha, B lymphocyte stimulator family of ligands and receptors, proliferation-inducing ligand, and identify of organ damage such as lupus nephritis, e.g., specific cytokines, chemokines, cell adhesion molecules, and growth and fibrosis factors [3, 10, 11]. However, the lack of clinical validation and the high processing and analytical costs associated with their determination represent a significant barrier to their introduction in routine practice [3].

Another group of biomarkers that is gaining increasing popularity in the area of autoimmune and autoinflammatory disorders, as well as other disease states such as atherosclerosis, cardiovascular disease, and cancer [12–14], is represented by hematological indices that are derived from routine blood cell counts, particularly neutrophils, platelets, and lymphocytes. These indexes are easy to determine and relatively inexpensive, factors that have facilitated their investigation in different disease states. Specifically, the neutrophil-to-lymphocyte ratio (NLR) and the platelet-to-lymphocyte ratio (PLR) have been shown to be significantly higher in patients with a range of immunological diseases when compared to healthy controls [15–20]. Although similar elevations in the NLR and PLR have been reported in patients with SLE [21, 22], a comprehensive investigation of their diagnostic accuracy for the presence and the severity of SLE as well as specific organ involvement and complications is lacking.

Therefore, we sought to address this issue by conducting a systematic review and meta-analysis of studies reporting the sensitivity and specificity of the NLR and PLR, obtained by receiver operating characteristic (ROC) curve analysis, for the presence of SLE, disease severity, common organ involvement (i.e., lupus nephritis, pericarditis, and pleural disease) [23], and complications (infections) [24].

## Methods

### Literature search

Two investigators independently conducted a systematic literature search in the electronic databases, PubMed, Web of Science, and Scopus, from inception to 15 of March 2024, using the following terms: “systemic lupus erythematosus” or “SLE” and “neutrophil to lymphocyte ratio” or “neutrophil-to-lymphocyte ratio” or “NLR” or “platelet to lymphocyte ratio” or “platelet-to-lymphocyte ratio” or “PLR.” The inclusion criteria were the following: (i) Studies reporting diagnostic accuracy by sensitivity and specificity, obtained by receiver operating characteristic (ROC) curve analysis, for the presence of SLE, disease severity, organ involvement (i.e., lupus nephritis, pericarditis, and pleural disease), and complications (infections); (ii) participants aged ≥ 18 years, (iii) articles in English language, and (iv) full text of the publication available. We hand searched the reference lists of each article to identify additional studies.

Data extracted included age, sex, publication year, study design, geographic area where the study was performed, sample size, area under the receiver operating characteristic curve (AUROC) with 95% confidence intervals (CIs), sensitivity, specificity, and cut-off values used for the NLR and PLR. True positive (TP), false positive (FP), false negative (FN), and true negative (TN) values were either extracted or calculated by generating 2 × 2 tables considering that sensitivity and specificity are derived from the following formulas: Sensitivity = TP/(TP + FN); Specificity = TN/(FP + TN) [25].

The risk of bias was assessed using the Joanna Briggs Institute Critical Appraisal Checklist for case–control studies [26]. The study adhered to the PRISMA 2020 statement on the reporting of systematic reviews and meta-analyses (PRISMA checklist in Supplementary Table 1) [27]. The protocol was registered in an international repository (PROSPERO registration number: CRD42024531446).

### Statistical analysis

Forest plots of the pooled sensitivity and specificity were generated to assess the diagnostic performance of the NLR and PLR for the presence of SLE, disease severity, organ involvement, and complications [28]. Summary receiving characteristics (SROC) curves with 95% confidence region and prediction region were generated using the MIDAS command [28]. To estimate the influence of each data point on the overall results of the meta-analysis and identify outliers, Cook’s distance measurement was performed [29]. The relationship between prior probability, likelihood ratio, and posterior test probability was assessed using the Fagan’s Nomogram plot [30]. All analyses were conducted using Stata 14 (StataCorp LLC, College Station, TX, USA).

## Results

### Study selection

The search strategy led to the identification of 509 articles of which 473 were excluded because they either presented duplicate or irrelevant data. A comprehensive review of the remaining 36 articles led to the further exclusion of nine (missing data, *n* = 6, non-adult participants, *n* = 3), leaving 27 studies for further analysis [31–57] (Supplementary Fig. 1). The risk of bias was ranked as low in all studies except one, which exhibited moderate risk [35] (Supplementary Table 2).

### Neutrophil-to-lymphocyte ratio

#### Presence of systemic lupus erythematosus

Eight studies investigating a total of 2,171 subjects (1,123 SLE patients and 1,021 healthy controls, 85% females, mean age 31.6 years) reported the sensitivity and specificity of the NLR for the presence of SLE [31–33, 38, 43, 45, 47, 56] (Supplementary Table 3). Five studies were conducted in China [32, 33, 38, 43, 47], one in Indonesia [31], one in Iran [45], and one in Turkey [56]. Six studies were retrospective [32, 33, 38, 43, 47, 56], and two prospective [31, 45].

**Fig. 1:**
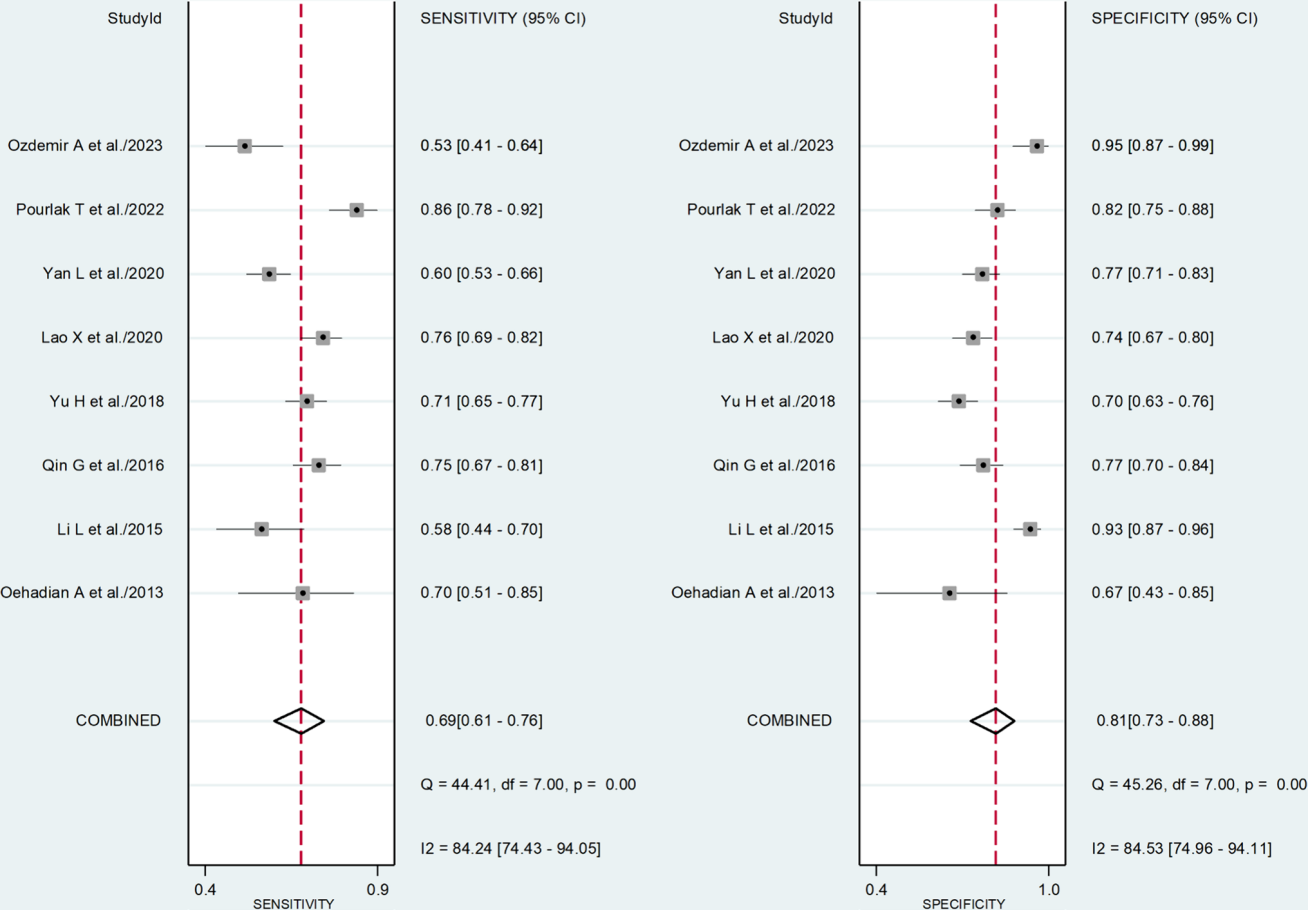
Forest plot of the pooled estimates of sensitivity and specificity of the neutrophil-to-lymphocyte ratio for the presence of systemic lupus erythematosus

**Fig. 2:**
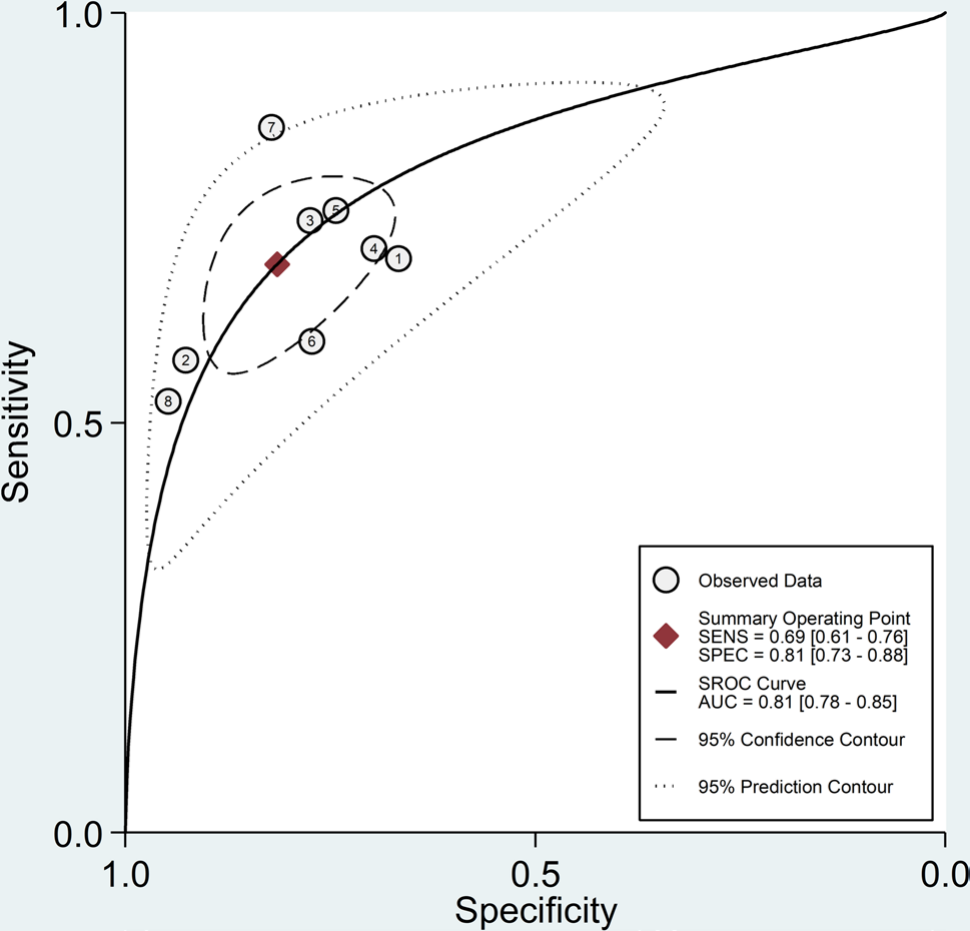
Summary receiver operating characteristics curve with 95% confidence region and prediction region of the neutrophil-to-lymphocyte ratio for the presence of systemic lupus erythematosus

The pooled sensitivity and specificity of the NLR for the presence of SLE were 0.69 (95% CI 0.61–0.76) and 0.81 (95% CI 0.73–0.88), respectively (Fig. 1). The SROC curve with 95% confidence region and prediction region is presented in Fig. 2. The AUC value was 0.81 (95% CI 0.78–0.85), with the summary operating point at sensitivity of 0.69 and specificity of 0.81. Assessment of publication bias and meta-regression could not be performed because of the small number of studies.

The Fagan’s nomogram showed that, assuming a pre-test probability of SLE of 25%, the post-test probability was 56% in subjects with relatively higher NLR values and 11% in those with relatively lower NLR values.

#### Disease severity

Nine studies assessing a total of 1,305 SLE patients (72% females, mean age 35 years) reported the sensitivity and specificity of the NLR for severe disease [34, 37, 40–42, 46, 51, 53, 57] (Supplementary Table 4). Three studies were performed in Egypt [41, 46, 53], three in China [34, 40, 57], one in Indonesia [42], one in Iraq [37], and one in Spain [51]. Six studies were retrospective [34, 40, 42, 46, 51, 57]; while, three were prospective [37, 41, 53]. Disease severity was evaluated using the Systemic Lupus Erythematosus Disease Activity Index [58] in five studies [40–42, 46, 53] and the Systemic Lupus Erythematosus Disease Activity Index 2000 [58] in three [34, 51, 57]. No information regarding the tool used to assess disease severity was provided in the remaining study [37]. The threshold for disease severity was set at 4 in four studies [41, 42, 46, 51], 6 in one study [53], and 9 in the remaining three studies [34, 40, 57]. One study did not report the threshold for severity [37].

The pooled sensitivity and specificity of the NLR for disease severity were 0.72 (95% CI 0.65–0.79) and 0.57 (95% CI 0.49–0.65), respectively (Supplementary Fig. 2). The SROC curve with 95% confidence region and prediction region showed an AUC value of 0.69 (95% CI 0.65–0.73), with the summary operating point at sensitivity of 0.72 and specificity of 0.57. Assessment of publication bias and meta-regression analysis could not be performed because of the small number of studies.

The Fagan’s nomogram showed that, assuming a pre-test probability of 25%, the post-test probability was 36% in subjects with relatively high NLR values and 14% in those with relatively low NLR values.

#### Lupus nephritis

Nine studies investigating a total of 1,056 SLE patients (89% females, mean age 34.5 years) reported the sensitivity and specificity of the NLR for lupus nephritis [32, 33, 35, 45, 46, 54–57] (Supplementary Table 5). Four studies were conducted in China [32, 33, 54, 57], two in Turkey [35, 56], two in Egypt [46, 55], and one in Iran [45]. Seven studies were retrospective [32, 33, 35, 46, 54, 56, 57], and two prospective [45, 55].

**Fig. 3:**
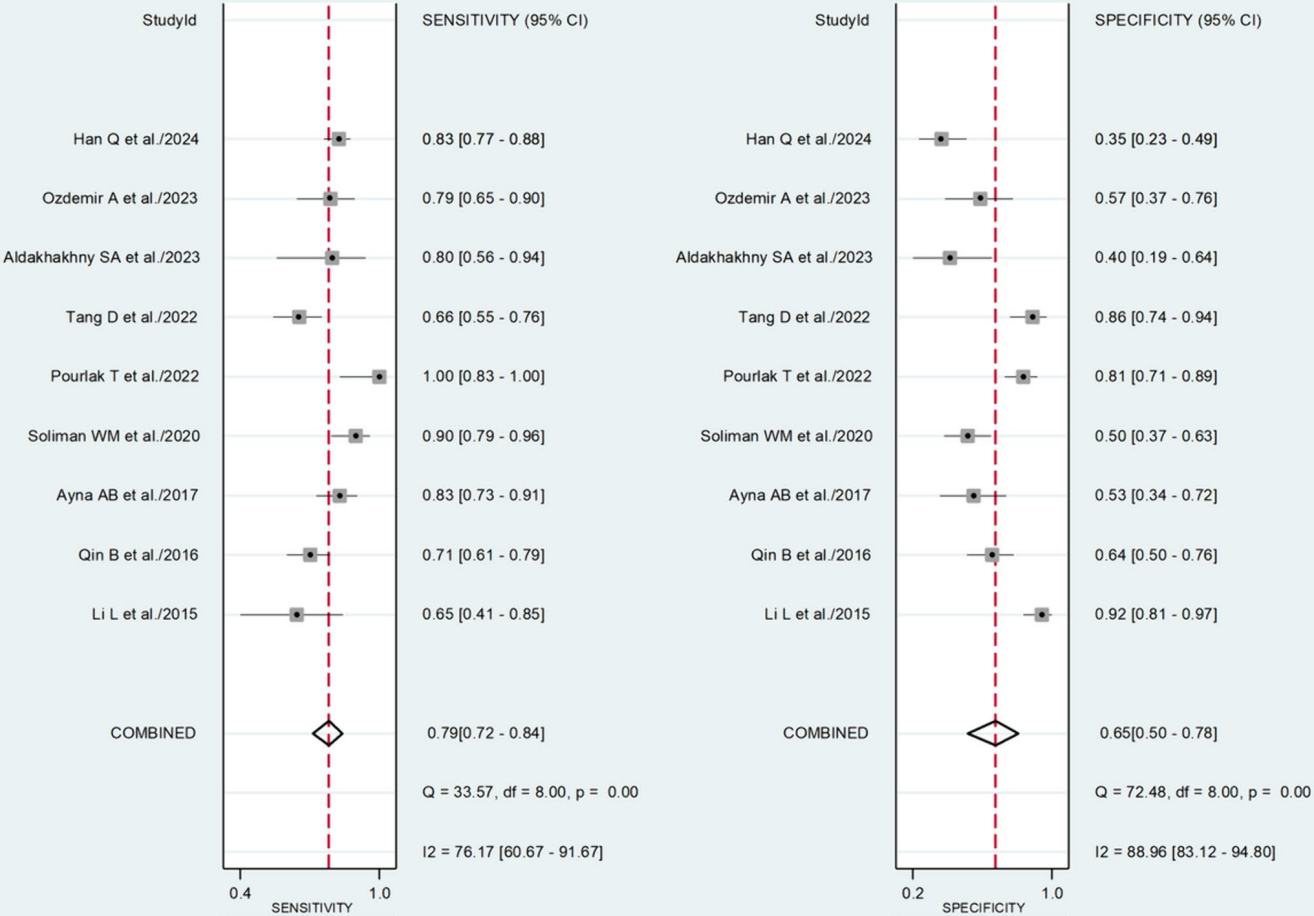
Forest plot for the pooled estimates of sensitivity and specificity of the neutrophil-to-lymphocyte ratio for lupus nephritis

**Fig. 4:**
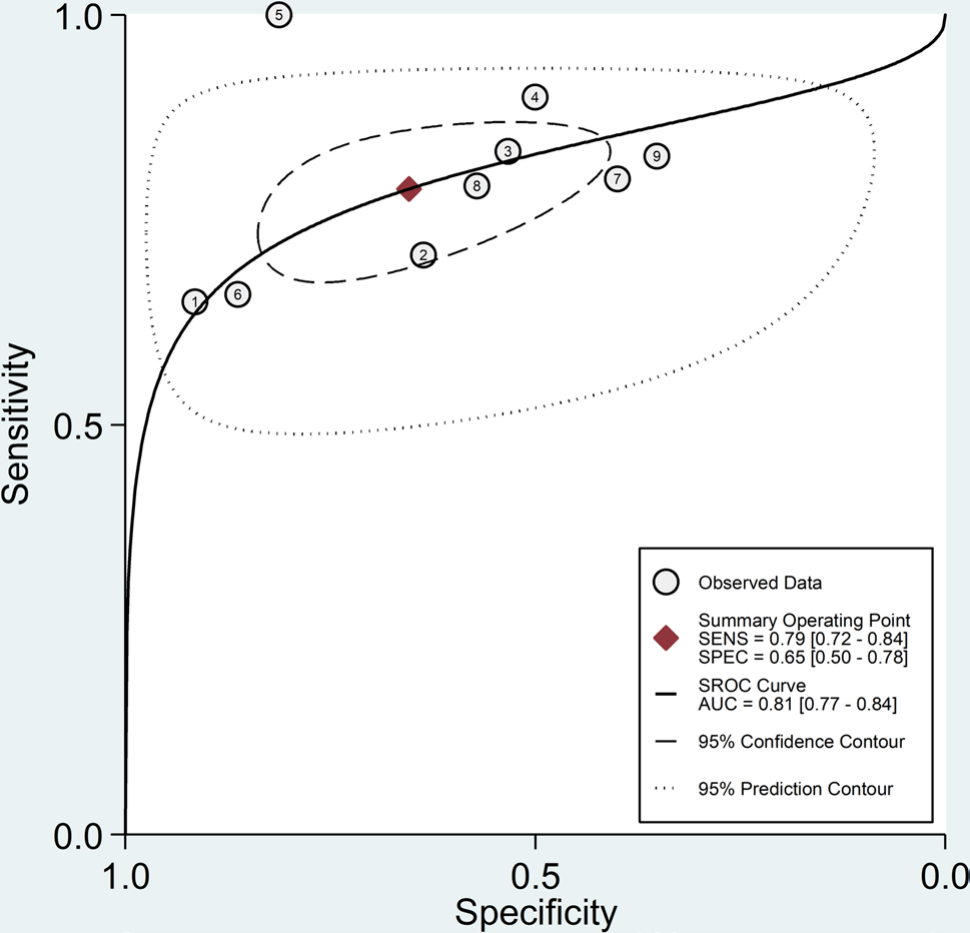
Summary receiver operating characteristics curve with 95% confidence region and prediction region of the neutrophil-to-lymphocyte ratio for lupus nephritis

The pooled sensitivity and specificity were 0.79 (95% CI 0.72–0.84) and 0.65 (95% CI 0.50–0.78), respectively (Fig. 3). The SROC curve with 95% confidence region and prediction region is presented in Fig. 4. The AUC value was 0.81 (95% CI 0.77–0.84), with the summary operating point at sensitivity of 0.79 and specificity of 0.65. Assessment of publication bias and meta-regression analysis could not be performed because of the small number of studies.

The Fagan’s nomogram showed that, assuming a pre-test probability of 25%, the post-test probability was 43% in patients with relatively high NLR values and 10% in those with relatively low NLR values.

#### Pericarditis and pleural disease

No studies investigating the sensitivity and specificity of the NLR for pericarditis or pleural disease were identified in our search.

#### Infections

Six studies investigating 690 SLE patients (86% females, mean age 34 years) reported the sensitivity and specificity of the NLR for infections [36, 39, 44, 48, 50, 52] (Supplementary Table 6). Two studies were conducted in India [50, 52], one in South Korea [36], one in Mexico [39], one in China [44], and one in Brazil [48]. Five studies were prospective [36, 39, 44, 50, 52], and one retrospective [48].

The pooled sensitivity and specificity were 0.67 (95% CI 0.60–0.74) and 0.75 (95% CI 0.63–0.84), respectively (Supplementary Fig. 3). The SROC curve with 95% confidence region and prediction region showed an AUC value of 0.73 (95% CI 0.69–0.77), with the summary operating point at sensitivity of 0.67 and specificity of 0.75. Assessment of publication bias and meta-regression analysis could not be performed because of the small number of studies.

The Fagan’s nomogram showed that, assuming a pre-test probability of 25%, the post-test probability was 47% in patients with relatively high NLR values and 13% in those with relatively low NLR values.

### Platelet-to-lymphocyte ratio

#### Presence of systemic lupus erythematosus

Three studies investigating 954 subjects (493 SLE patients and 461 healthy controls, 90% females, mean age 38 years) reported the sensitivity and specificity of the PLR for the presence of SLE [43, 47, 56] (Supplementary Table 3). Two studies were conducted in China [43, 47], and one in Turkey [56]. All three studies were retrospective. Forest plots for pooled sensitivity and specificity and the SROC curve could not be generated given the limited number of studies. Lao et al. reported an AUC value of 0.72 with 0.738 sensitivity and 0.684 specificity [43]. Yan et al. reported an AUC of 0.577 with 0.342 sensitivity and 0.896 specificity [47]. Ozdemir et al. reported an AUC of 0.666 with 0.565 sensitivity and 0.742 specificity [56].

#### Disease severity

Six studies investigating 711 SLE patients (86% females, mean age 35 years) reported the sensitivity and specificity of the PLR for severe disease [34, 41, 46, 49, 51, 53] (Supplementary Table 4). Four studies were conducted in Egypt [41, 46, 49, 53], one in China [34], and one in Spain [51]. Three studies were retrospective [34, 46, 51], and three prospective [41, 49, 53]. The Systemic Lupus Erythematosus Disease Activity Index [58] was used in in three studies [41, 46, 53] and the Systemic Lupus Erythematosus Disease Activity Index 2000 [58] in two [34, 51]. One study did not report information regarding the tool used to assess severity [49]. The severity threshold was set at 4 in three studies [41, 46, 51], 6 in one study [53], and 9 in another study [34].

**Fig. 5:**
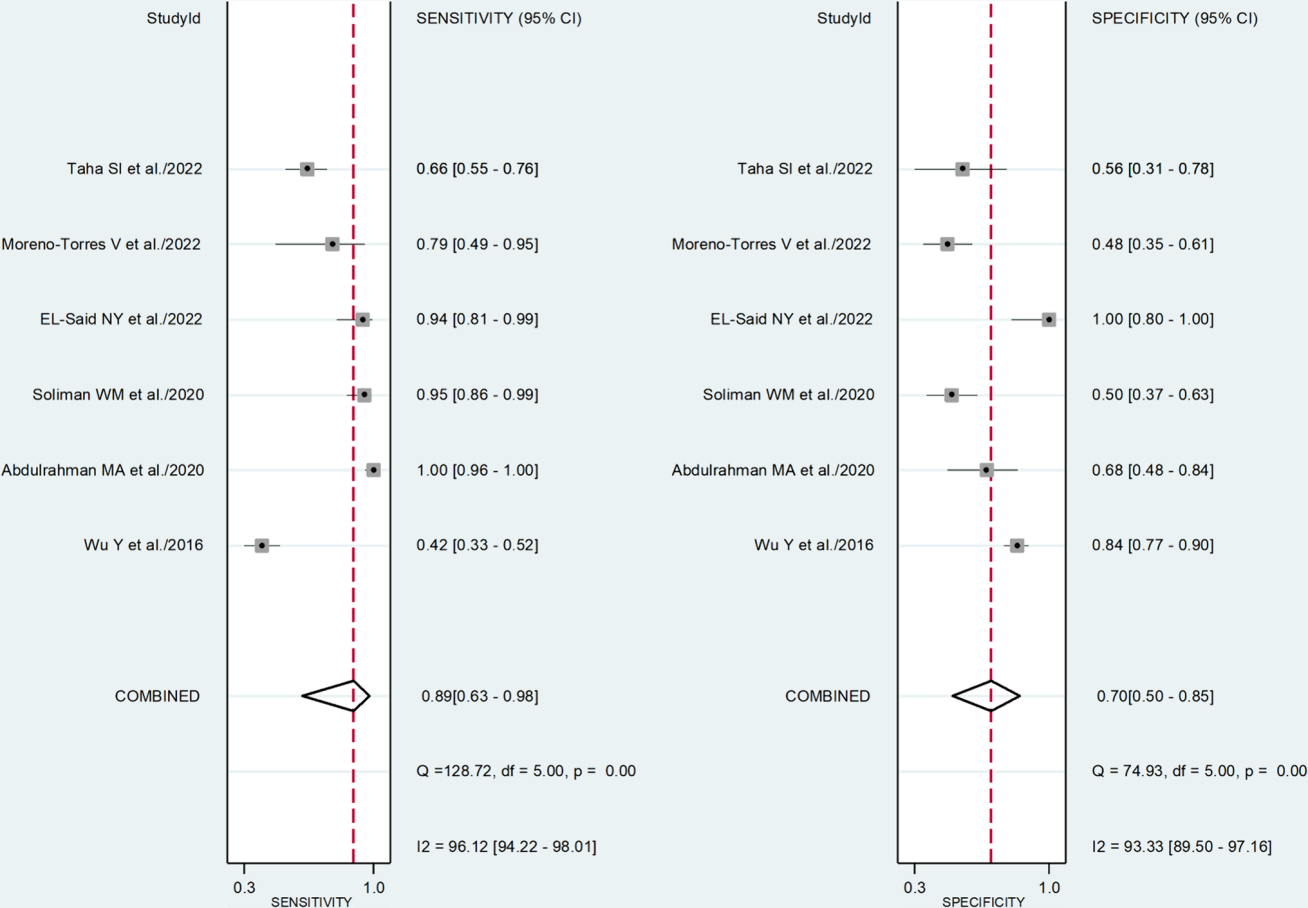
Forest plot of the pooled estimates of sensitivity and specificity of the platelet-to-lymphocyte ratio for severe disease

**Fig. 6:**
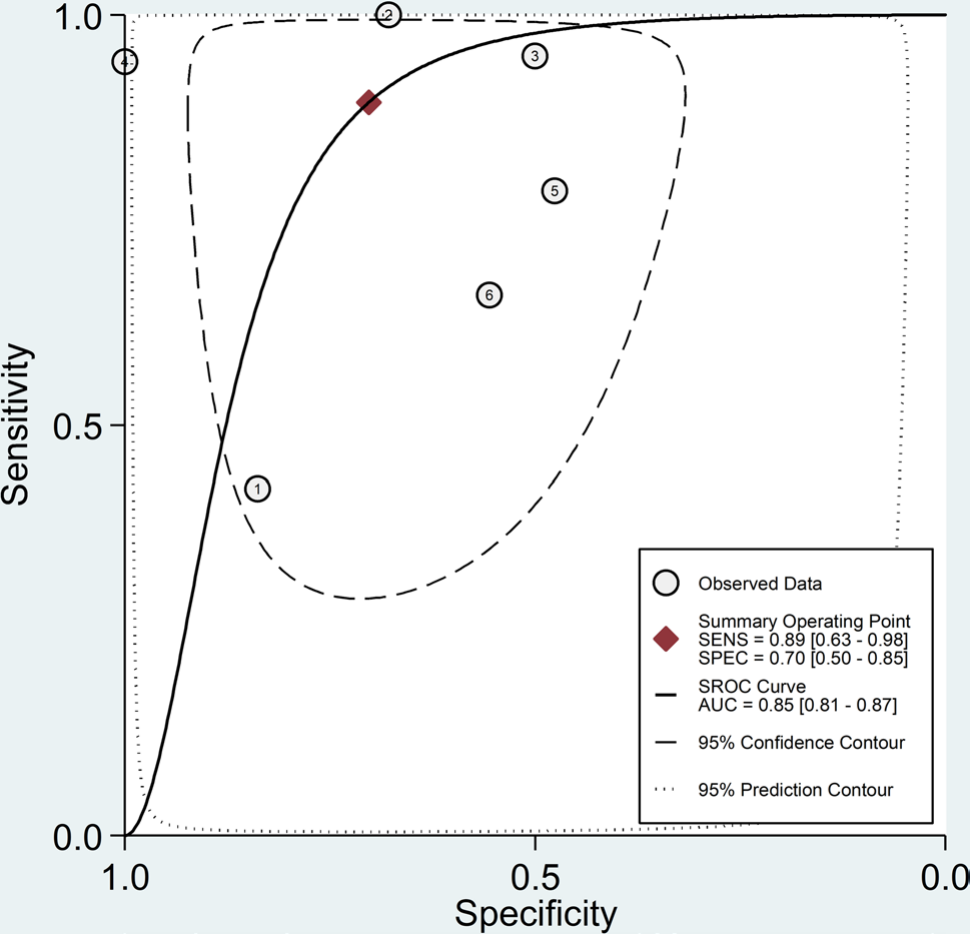
Summary receiver operating characteristics curve with 95% confidence region and prediction region of the platelet-to-lymphocyte ratio for severe disease

The pooled sensitivity and specificity were 0.89 (95% CI 0.63–0.98) and 0.70 (95% CI 0.50–0.85), respectively (Fig. 5). The SROC curve with 95% confidence region and prediction region is illustrated in Fig. 6. The AUC value was 0.85 (95% CI 0.81–0.87), with the summary operating point at sensitivity of 0.89 and specificity of 0.70. Assessment of publication bias and meta-regression analysis could not be performed because of the small number of studies.

The Fagan’s nomogram showed that, assuming a pre-test probability of 25%, the post-test probability was 50% in patients with relatively high NLR values and 5% in those with relatively low NLR values.

#### Lupus nephritis

One prospective study conducted in Egypt in 40 SLE patients (98% females, mean age 37 years) reported the sensitivity and specificity of the PLR for lupus nephritis [55] (Supplementary Table 5). The AUC was 0.69 with values of 0.7 and 0.6 for sensitivity and specificity, respectively.

#### Pericarditis and pleural disease

No studies investigating the sensitivity and specificity of the PLR for pericarditis or pleural disease were identified in our search.

#### Infections

Two studies investigating 256 SLE patients (86% females, mean age 30 years) reported the sensitivity and specificity of the PLR for infections [48, 52] (Supplementary Table 6). One study was retrospective and conducted in Brazil [48], and the other was prospective and conducted in India [52]. Forest plots for pooled sensitivity and specificity and the SROC curve could not be generated due to the limited number of studies. Carvalho et al. reported a sensitivity of 0.71 and specificity of 0.53 [48]; whereas, Musunuri et al. reported a sensitivity of 0.676 and specificity of 0.689 [52].

## Discussion

The results of this systematic review and meta-analysis suggest that hematological indices that are easily derived from routine full blood cell counts can be useful in diagnosing specific features in patients with SLE. In particular, based on the observed AUC values [59, 60], the NLR was shown to have a good diagnostic accuracy for the presence of SLE and organ involvement, i.e., lupus nephritis; whereas, the PLR exhibited a good diagnostic accuracy for the presence of severe disease. These observations support the potential clinical application of the NLR and the PLR, in combination with clinical evaluation and available serological biomarkers, in the diagnosis and management of patients with SLE. This proposition is further supported by the Fagan’s nomogram, which showed a good separation in the probability of having SLE, lupus nephritis, and severe disease given a relatively high/low NLR (presence of SLE and lupus nephritis) and PLR (presence of severe disease). However, further research is required to confirm our findings and to investigate the diagnostic accuracy of the NLR and PLR for other clinical manifestations associated with the disease, e.g., cardiac, pulmonary, and neuropsychiatric abnormalities, as well as the effects of treatment. The results of these studies will be instrumental to justify the use of the NLR and PLR in the routine assessment of patients with SLE.

Previous investigations have assessed the diagnostic accuracy of currently used serological biomarkers in SLE. In one study of 304 SLE patients, 285 patients with other rheumatic diseases, and 205 healthy individuals, the AUC for the presence of SLE was 0.79 ± 0.02 with antibodies to double-stranded DNA, 0.73 ± 0.02 with complement C3, and 0.72 ± 0.02 with complement C4 [61]. In another study, the AUC for the presence of lupus renal flare in 71 patients was 0.74 for complement C3 and 0.65 for complement C4, respectively [62]. Other studies have reported AUC values for lupus nephritis ranging between 0.65 and 0.94 with antibodies to double-stranded DNA, 0.99 with proteinuria (> 500 mg/24 h), and 0.83 with kidney biopsy [10]. The AUC values observed in our systematic review and meta-analysis for the diagnosis of SLE (NLR: 0.81), lupus nephritis (NLR: 0.81), and severe disease (PLR: 0.85) are comparable or higher than those reported with available serological biomarkers. However, further research is warranted to investigate whether measuring the NLR and PLR can effectively complement the already high sensitivity and specificity observed with the 2019 EULAR/ACR criteria, the 2012 SLICC criteria, and the 1997 ACR criteria [6].

There is good evidence that neutrophils, platelets, and lymphocytes play a critical role in the pathophysiology of SLE. However, the reported elevations in the NLR and PLR in this group do not necessarily reflect an absolute increase in the neutrophil and platelet count. In fact, a state of neutropenia, reported in 20–40% of SLE patients, is attributed to several causes, including immune-mediate destruction, disease activity, splenomegaly, viral infection, and the use of immunosuppressive drugs [63, 64]. Similarly, thrombocytopenia has been reported in 20–50% of SLE patients and is commonly caused by immune-mediated destruction, and less frequently by the use of specific drugs, splenomegaly, and increased platelet consumption in the presence of thrombotic micro-angiopathic processes or antiphospholipid syndrome [64]. By contrast, the lymphocyte count, the denominator in both the NLR and PLR, is negatively affected in SLE with a reported incidence of lymphocytopenia between 20–75%, likely secondary to the presence of specific autoantibodies to lymphocytes [63, 65, 66]. Further studies are therefore required to determine whether the ratio between specific cell types provides a more accurate diagnostic information when compared to individual cell populations in SLE.

Regardless of the actual cell count, an excessive neutrophil activation is involved in the release of reactive oxygen species and granule proteases in vascular and other tissues and in the post-translational modification of cell components, i.e., proteins and DNA [67, 68]. The release of specific cytokines and chemokines by this cell type regulates both the innate and adaptive immune responses in SLE, and neutrophil extracellular traps expose nuclear proteins to the immune system, initiating the production of autoantibodies (anti-dsDNA and anti-acetylated/methylated histones) [69]. Neutrophil apoptosis is also dysregulated in SLE, leading to increased apoptotic burden associated with development of anti-nuclear autoantibodies [70, 71]. Similarly, platelet activation is also observed in SLE and involves several molecular pathways including immune complexes, Toll-like receptors activation, antiphospholipid antibodies and ischemia–reperfusion associated with the Raynaud phenomenon, a common complication in these patients [72, 73]. Activated platelets can also dysregulate the immune system by priming interferon production, providing CD40L supporting B lymphocyte functions and providing a source of autoantigens, and favor end-organ damage and tissue fibrosis. Further studies are therefore warranted to investigate the interplay between alterations in neutrophil, platelet, lymphocyte count, modifications in the NLR and PLR, and functional alterations of neutrophil and platelets in SLE patients with different disease severity, organ involvement, and clinical progression.

A strength of our systematic review and meta-analysis is the comprehensive assessment of published studies reporting the sensitivity and specificity of the NLR and PLR in SLE and various clinical manifestations, which provides important information regarding the diagnostic accuracy of these tools, assessed using the AUC and the Fagan’s nomogram. One important limitation is represented by the limited number of studies conducted in Europe and North and South America, which limits the generalizability of our findings. This issue is particularly important as ethnic-related differences in the NLR have been reported in subjects without autoimmune diseases [74–77]. Another limitation is the lack of information, in the selected studies, regarding the possible impact of additional factors on the diagnostic performance of these hematological indices. Such factors are common in SLE patients and include atherosclerosis and cardiovascular disease [78, 79], viral and bacterial infections [80, 81], and malignancies [82], as well as immunosuppressive therapies [83, 84].

In conclusion, our study suggests that the NLR and the PLR are promising biomarkers for the diagnosis of SLE, severe disease, and presence of specific organ damage. Adequately designed prospective studies are warranted to determine whether these hematological indices can significantly enhance the diagnostic accuracy of clinical assessment, existing SLE classification criteria and serological biomarkers independently of other factors known to influence the NLR and the PLR.

## Supplementary Information

Below is the link to the electronic supplementary material.

Supplementary file1 (TIFF 4016 KB)

Supplementary file2 (TIFF 4591 KB)

Supplementary file3 (TIFF 3929 KB)

Supplementary file4 (DOCX 15 KB)

Supplementary file5 (DOCX 34 KB)

Supplementary file6 (DOCX 50 KB)

Supplementary file7 (DOCX 26 KB)

Supplementary file8 (DOCX 30 KB)

Supplementary file9 (DOCX 28 KB)

Supplementary file10 (DOCX 26 KB)

## Data Availability

The data that support the findings of this systematic review and meta-analysis are available from AZ upon reasonable request.
