# Supplementary figures and images for: A systematic review and meta-analysis of the diagnostic accuracy of the neutrophil-to-lymphocyte ratio and the platelet-to-lymphocyte ratio in systemic lupus erythematosus
Source: Clin Exp Med. 2024 Jul 25;24(1):170. doi: 10.1007/s10238-024-01438-5 (PMC11272706; doi:10.1007/s10238-024-01438-5)

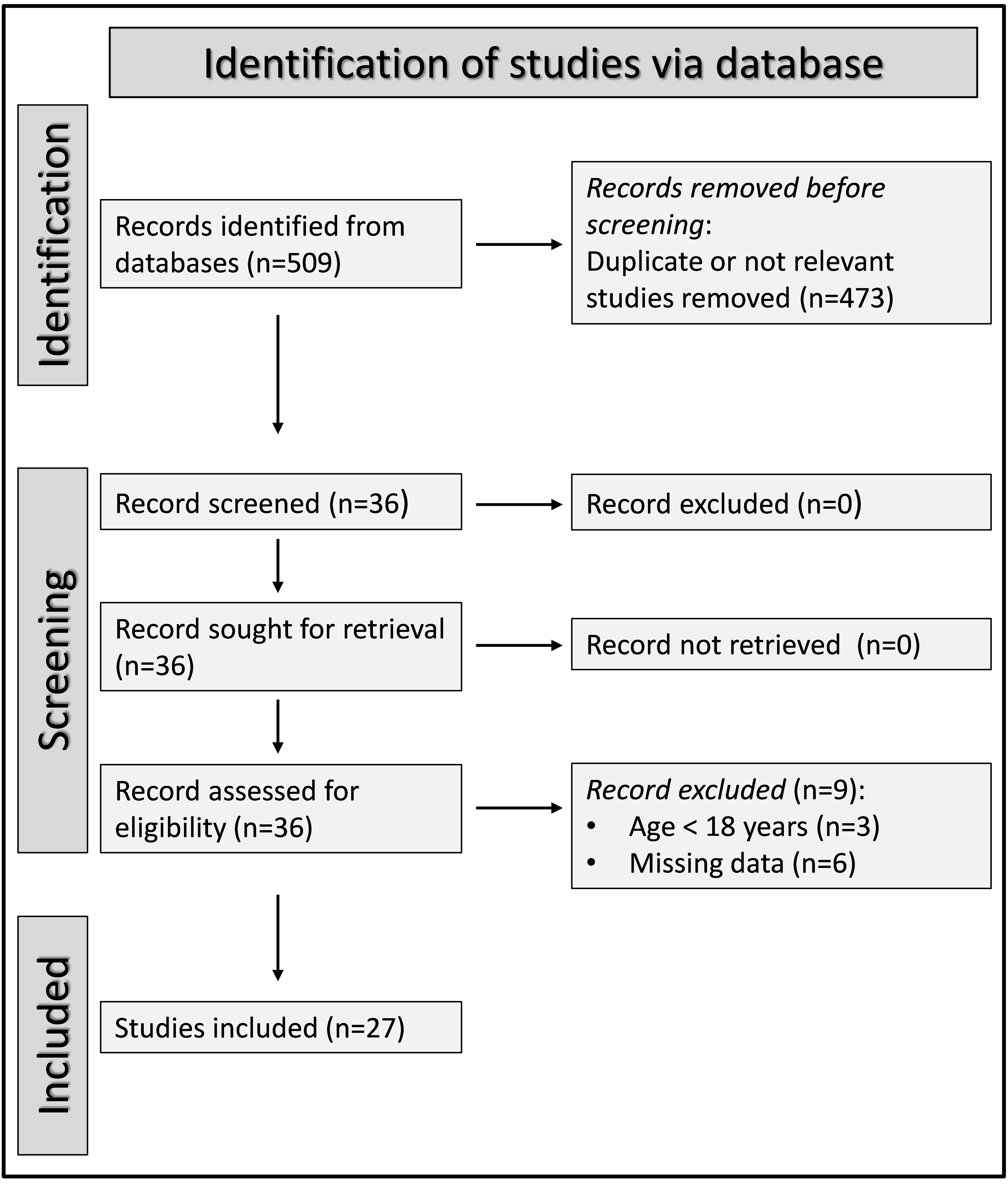

Supplement: Supplementary file 1 — Supplementary file1 (TIFF 4016 KB) [file 10238_2024_1438_MOESM1_ESM.tiff]

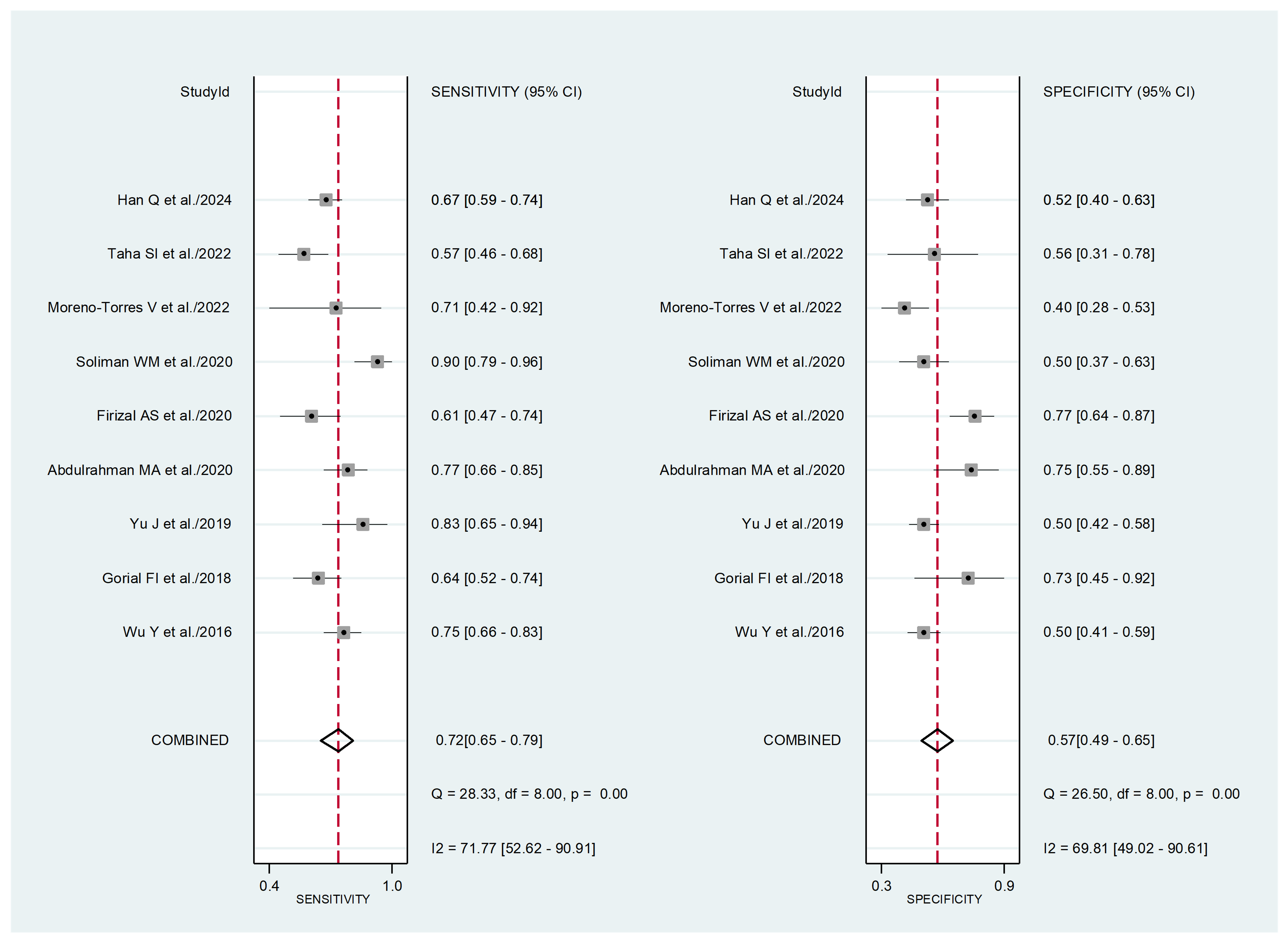

Supplement: Supplementary file 2 — Supplementary file2 (TIFF 4591 KB) [file 10238_2024_1438_MOESM2_ESM.tiff]

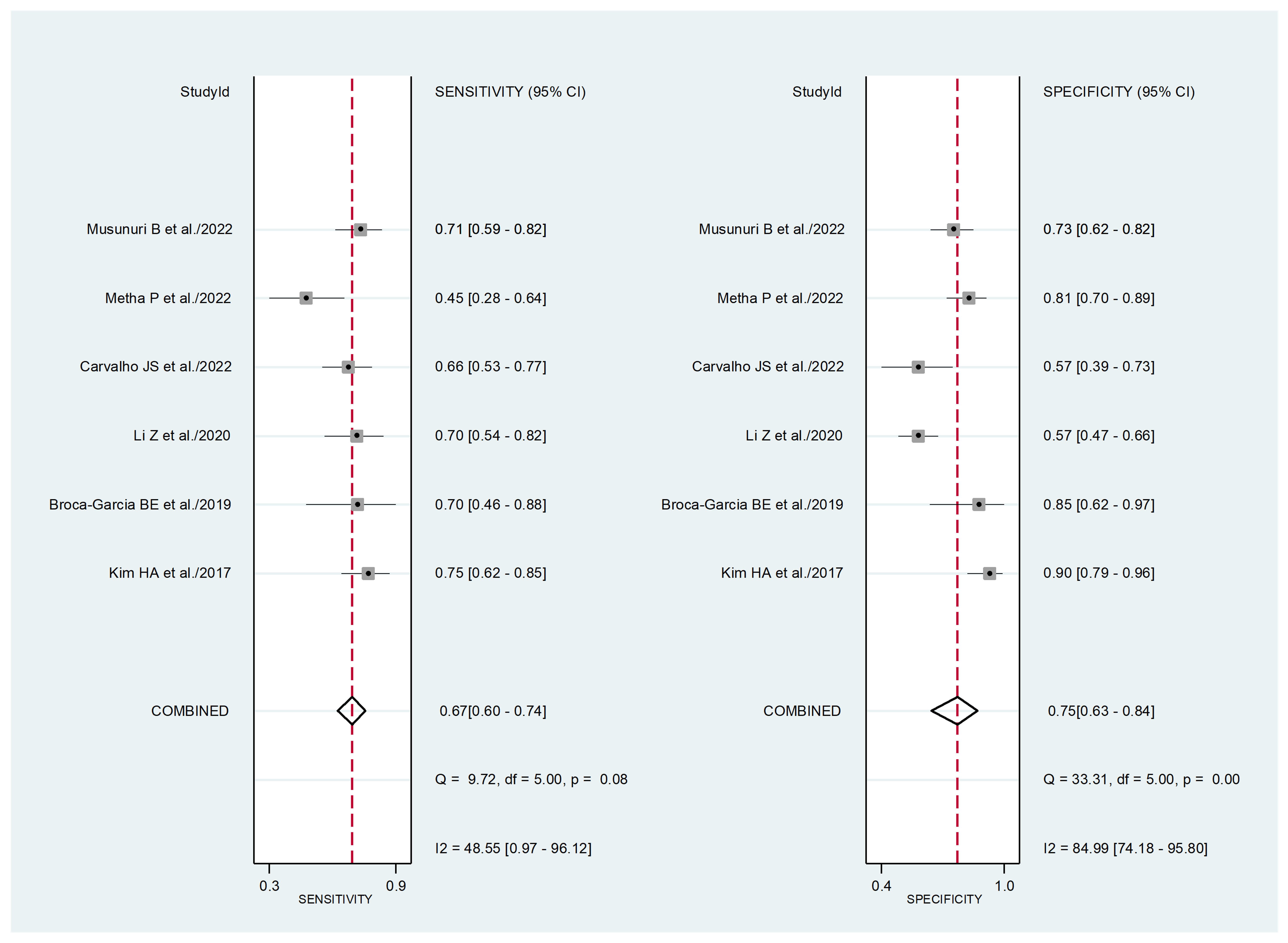

Supplement: Supplementary file 3 — Supplementary file3 (TIFF 3929 KB) [file 10238_2024_1438_MOESM3_ESM.tiff]
