# Supplementary material for: A systematic review and meta-analysis of the diagnostic accuracy of the neutrophil-to-lymphocyte ratio and the platelet-to-lymphocyte ratio in systemic lupus erythematosus
Source: Clin Exp Med. 2024 Jul 25;24(1):170. doi: 10.1007/s10238-024-01438-5 (PMC11272706; doi:10.1007/s10238-024-01438-5)
Supplement: Supplementary file 4 — Supplementary file4 (DOCX 15 KB) [file 10238_2024_1438_MOESM4_ESM.docx]

**Supplementary figure legends**

**Supplementary Figure 1.** Flow chart of study selection.

**Supplementary Figure 2.** Forest plot for the pooled estimates of sensitivity and specificity of the neutrophil-to-lymphocyte ratio for severe disease.

**Supplementary Figure 3.** Forest plot for the pooled estimates of sensitivity and specificity of the neutrophil-to-lymphocyte ratio for infection.
