# Supplementary material for: A systematic review and meta-analysis of the diagnostic accuracy of the neutrophil-to-lymphocyte ratio and the platelet-to-lymphocyte ratio in systemic lupus erythematosus
Source: Clin Exp Med. 2024 Jul 25;24(1):170. doi: 10.1007/s10238-024-01438-5 (PMC11272706; doi:10.1007/s10238-024-01438-5)
Supplement: Supplementary file 6 — Supplementary file6 (DOCX 50 KB) [file 10238_2024_1438_MOESM6_ESM.docx]

**Supplementary Table 2.** Assessment of the risk of bias using the Joanna Briggs Institute critical appraisal checklist.

| **Study** | **Were the inclusion criteria clearly defined?** | **Were the subjects and the setting described in detail?** | **Was the exposure measured in a reliable way?** | **Were standard criteria used to assess the condition?** | **Were confounding factors identified?** | **Were strategies to deal with confounding factors stated?** | **Were the outcomes measured in a reliable way?** | **Was appropriate statistical analysis used?** | **Risk of bias** |
| --- | --- | --- | --- | --- | --- | --- | --- | --- | --- |
| Oehadian A et al. [28] | Yes | Yes | Yes | Yes | No | No | Yes | Yes | Low |
| Li L et al. [29] | Yes | Yes | Yes | Yes | Yes | Yes | Yes | Yes | Low |
| Qin B et al. [30] | Yes | Yes | Yes | Yes | Yes | Yes | Yes | Yes | Low |
| Wu Y et al. [31] | Yes | Yes | Yes | Yes | No | No | Yes | Yes | Low |
| Ayna AB et al. [32] | No | Yes | Yes | Yes | No | No | Yes | Yes | Moderate |
| Kim HA et al. [33] | No | Yes | Yes | Yes | Yes | Yes | Yes | Yes | Low |
| Gorial FI et al. [34] | Yes | Yes | Yes | Yes | Yes | Yes | Yes | Yes | Low |
| Yu H et al [35] | Yes | Yes | Yes | Yes | Yes | Yes | Yes | Yes | Low |
| Broca-Garcia BE et al [36] | Yes | Yes | Yes | Yes | No | No | Yes | Yes | Low |
| Yu J et al. [37] | Yes | Yes | Yes | Yes | No | No | Yes | Yes | Low |
| Abdulrahman MA et al. [38] | Yes | Yes | Yes | Yes | No | No | Yes | Yes | Low |
| Firizal As et al. [39] | Yes | Yes | Yes | Yes | No | No | Yes | Yes | Low |
| Lao X et al. [40] | Yes | Yes | Yes | Yes | Yes | Yes | Yes | Yes | Low |
| Li Z et al. [41] | Yes | Yes | Yes | Yes | Yes | Yes | Yes | Yes | Low |
| Pouriak T et al. [42] | Yes | Yes | Yes | Yes | No | No | Yes | Yes | Low |
| Soliman WM et al. [43] | Yes | Yes | Yes | Yes | No | No | Yes | Yes | Low |
| Yan L et al. [44] | Yes | Yes | Yes | Yes | Yes | Yes | Yes | Yes | Low |
| Carvalho JS et al. [45] | Yes | Yes | Yes | Yes | Yes | Yes | Yes | Yes | Low |
| El-Said NY et al. [46] | Yes | Yes | Yes | Yes | No | No | Yes | Yes | Low |
| Metha P et al. [47] | Yes | Yes | Yes | Yes | Yes | Yes | Yes | Yes | Low |
| Moreno-Torres V et al. [48] | No | Yes | Yes | Yes | Yes | Yes | Yes | Yes | Low |
| Musunuri B et al. [49] | Yes | Yes | Yes | Yes | No | No | Yes | Yes | Low |
| Taha SI et al. [50] | Yes | Yes | Yes | Yes | Yes | Yes | Yes | Yes | Low |
| Tang D et al. [51] | Yes | Yes | Yes | Yes | No | No | Yes | Yes | Low |
| Aldakhakhny SA et al. [52] | Yes | Yes | Yes | Yes | No | No | Yes | Yes | Low |
| Ozdemir A et al. [53] | Yes | Yes | Yes | Yes | No | No | Yes | Yes | Low |
| Han Q et al. [54] | Yes | Yes | Yes | Yes | No | No | Yes | Yes | Low |
