# Supplementary material for: A systematic review and meta-analysis of the diagnostic accuracy of the neutrophil-to-lymphocyte ratio and the platelet-to-lymphocyte ratio in systemic lupus erythematosus
Source: Clin Exp Med. 2024 Jul 25;24(1):170. doi: 10.1007/s10238-024-01438-5 (PMC11272706; doi:10.1007/s10238-024-01438-5)
Supplement: Supplementary file 7 — Supplementary file7 (DOCX 26 KB) [file 10238_2024_1438_MOESM7_ESM.docx]

**Supplementary Table 3.** Summary of studies investigating diagnostic accuracy of the neutrophil-to-lymphocyte ratio and the platelet-to-lymphocyte ratio for the presence of systemic lupus erythematosus.

| **Study** | **Study design** | **N** | **Age (years)** | **M/F** | **AUC (95% CI)**  **NLR**  **PLR** | **Cut-off**  **NLR**  **PLR** | **Sensitivity (%)**  **NLR**  **PLR** | **Specificity (%)**  **NLR**  **PLR** |
| --- | --- | --- | --- | --- | --- | --- | --- | --- |
| Oehadian A et al. 2013, Indonesia [28] | P | 51 | 26 | 10/41 | 0.727 (NR)  NR | 1.93  NR | 0.7  NR | 0.67  NR |
| Li L et al. 2015, China [29] | R | 208 | 29 | 21/187 | 0.757 (0.668-0.845)  NR | 3.13  NR | 0.574  NR | 0.926  NR |
| Qin G et al. 2016, China [30] | R | 305 | 42 | 37/268 | 0.828 (0.781-0.785)  NR | 2.065  NR | 0.747  NR | 0.775  NR |
| Yu H et al. 2018, China [35] | R | 413 | 40 | 43/370 | 0.735 (0.684-0.786)  NR | 2.075  NR | 0.7114  NR | 0.6957  NR |
| Lao X et al. 2020, China [40] | R | 378 | NR | NR | 0.79 (NR)  0.72 (NR) | 1.98  145.64 | 0.758  0.738 | 0.744  0.684 |
| Pourlak T et al. 2022, Iran [42] | P | 240 | 37 | 38/202 | 0.904 (0.857-0.94)  NR | 1.98  NR | 0.862  NR | 0.82  NR |
| Yan L et al. 2020, China [44] | R | 424 | 42 | 38/377 | 0.706 (0.657-0.756)  0.577 (0.522-0.633) | 2.68  203.17 | 0.6  0.342 | 0.77  0.896 |
| Ozdemir A et al. 2023, Turkey [53] | R | 152 | 33 | 76/76 | 0.723 (0.636-0.810)  0.666 (0.573-0.758) | 2.95  NR | 0.521  0.565 | 0.942  0.742 |

Legend: NR, not reported; P, prospective; R, retrospective; M/F, male to female ratio; AUC, area under the curve; NLR, neutrophil-to-lymphocyte ratio; PLR, platelet-to-lymphocyte ratio.
