# Supplementary material for: A systematic review and meta-analysis of the diagnostic accuracy of the neutrophil-to-lymphocyte ratio and the platelet-to-lymphocyte ratio in systemic lupus erythematosus
Source: Clin Exp Med. 2024 Jul 25;24(1):170. doi: 10.1007/s10238-024-01438-5 (PMC11272706; doi:10.1007/s10238-024-01438-5)
Supplement: Supplementary file 8 — Supplementary file8 (DOCX 30 KB) [file 10238_2024_1438_MOESM8_ESM.docx]

**Supplementary Table 4.** Summary of studies investigating diagnostic accuracy of the neutrophil-to-lymphocyte ratio and the platelet-to-lymphocyte ratio for the severity of systemic lupus erythematosus.

| **Study** | **Study design** | **N** | **Age (years)** | **M/F** | **AUC (95% CI)**  **NLR**  **PLR** | **Cut-off**  **NLR**  **PLR** | **Sensitivity (%)**  **NLR**  **PLR** | **Specificity (%)**  **NLR**  **PLR** |
| --- | --- | --- | --- | --- | --- | --- | --- | --- |
| Wu Y et al. 2016, China [31] | R | 252 | 27 | 141/111 | 0.64 (0.54-0.67)  0.613 (0.51-0.72) | 2.26  203.85 | 0.75  0.423 | 0.5  0.839 |
| Gorial FI et al. 2018, Iraq [34] | P | 100 | 32 | 9/91 | 0.7 (NR)  NR | 2.19  NR | 0.635  NR | 0.733  NR |
| Yu J et al. 2019, China [37] | R | 194 | 41 | 15/179 | 0.689 (0.578-0.800)  NR | 2.298  NR | 0.828  NR | 0.497  NR |
| Abdulrahman MA et al. 2020, Egypt [38] | P | 110 | 26 | 16/94 | NR  NR | 5.7  316.5 | 0.77  1.00 | 0.75  0.68 |
| Firizal AS et al. 2020, Indonesia [39] | R | 112 | 34 | 5/107 | 0.677 (0.577-0.777)  NR | 2.94  NR | 0.6071  NR | 0.7679  NR |
| Soliman WM et al. 2020, Egypt [43] | R | 120 | 30 | 18/102 | 0.709 (0.542-0.875)  0.762 (0.614-0.911) | 2.2  132.9 | 0.9  0.95 | 0.5  0.5 |
| El-Said NY et al. 2022, Egypt [46] | P | 52 | 33 | 6/46 | NR  0.69 (NR) | NR  33.6 | NR  0.943 | NR  1.00 |
| Moreno-Torres V et al. 2022, Spain [48] | R | 77 | 47 | 0/77 | 0.63 (NR)  0.68 (NR) | 1.97  154 | 0.69  0.77 | 0.4  0.47 |
| Taha SI et al. 2022, Egypt [50] | P | 100 | 65 | 16/84 | 0.418 (0.248-0.588)  0.638 (0.464-0.812) | 2.13  116.66 | 0.573  0.659 | 0.556  0.556 |
| Han Q et al. 2024, China [54] | R | 240 | 28 | 146/94 | 0.59 (0.51-0.66)  NR | 2.19  NR | 0.671  NR | 0.519  NR |

Legend: NR, not reported; P, prospective; R, retrospective; M/F, male to female ratio; AUC, area under the curve; NLR, neutrophil-to-lymphocyte ratio; PLR, platelet-to-lymphocyte ratio.
