# Supplementary material for: A systematic review and meta-analysis of the diagnostic accuracy of the neutrophil-to-lymphocyte ratio and the platelet-to-lymphocyte ratio in systemic lupus erythematosus
Source: Clin Exp Med. 2024 Jul 25;24(1):170. doi: 10.1007/s10238-024-01438-5 (PMC11272706; doi:10.1007/s10238-024-01438-5)
Supplement: Supplementary file 9 — Supplementary file9 (DOCX 28 KB) [file 10238_2024_1438_MOESM9_ESM.docx]

**Supplementary Table 5.** Summary of studies investigating diagnostic accuracy of the neutrophil-to-lymphocyte ratio and the platelet-to-lymphocyte ratio for the presence of lupus nephritis in patients with systemic lupus erythematosus.

| **Study** | **Study design** | **N** | **Age (years)** | **M/F** | **AUC (95% CI)**  **NLR**  **PLR** | **Cut-off**  **NLR**  **PLR** | **Sensitivity (%)**  **NLR**  **PLR** | **Specificity (%)**  **NLR**  **PLR** |
| --- | --- | --- | --- | --- | --- | --- | --- | --- |
| Li L et al. 2015, China [29] | R | 79 | 28 | 6/73 | 0.828 (0.723-0.932)  NR | 4.4  NR | 0.647  NR | 0.916  NR |
| Qin B et al. 2016, China [30] | R | 154 | 41 | 17/137 | 0.715 (0.616-0.787)  NR | 2.664  NR | 0.707  NR | 0.636  NR |
| Ayna AB et al. 2017, Turkey [32] | R | 108 | 35 | 8/100 | 0.76 (0.66-0.82)  NR | 1.93  NR | 0.83  NR | 0.54  NR |
| Pourlak T et al. 2022, Iran [42] | P | 100 | 36 | 14/86 | 0.935 (0.867-0.974)  NR | 3.64  NR | 1  NR | 0.8125  NR |
| Soliman WM et al. 2020, Egypt [43] | R | 120 | 30 | 18/102 | 0.747 (0.594-0.901)  NR | 2.2  NR | 0.9  NR | 0.5  NR |
| Tang D et al. 2022, China [51] | R | 139 | 45 | 20/119 | 0.785 (0.708-0.862)  NR | 5.44  NR | 0.659  NR | 0.863  NR |
| Aldakhakhny SA et al. 2023, Egypt [52] | P | 40 | 37 | 1/39 | 0.73 (NR)  0.69 (NR) | 3.6  186 | 0.8  0.7 | 0.4  0.6 |
| Ozdemir A et al. 2023, Turkey [53] | R | 76 | 33 | 10/66 | 0.635 (0.509-0.762)  NR | 2.32  NR | 0.785  NR | 0.562  NR |
| Han Q et al. 2024, China [54] | R | 240 | 28 | 21/219 | 0.59 (0.5-0.68)  NR | 1.71  NR | 0.828  NR | 0.352  NR |

Legend: NR, not reported; P, prospective; R, retrospective; M/F, male to female ratio; AUC, area under the curve; NLR, neutrophil-to-lymphocyte ratio; PLR, platelet-to-lymphocyte ratio.
