# Supplementary material for: A systematic review and meta-analysis of the diagnostic accuracy of the neutrophil-to-lymphocyte ratio and the platelet-to-lymphocyte ratio in systemic lupus erythematosus
Source: Clin Exp Med. 2024 Jul 25;24(1):170. doi: 10.1007/s10238-024-01438-5 (PMC11272706; doi:10.1007/s10238-024-01438-5)
Supplement: Supplementary file 10 — Supplementary file10 (DOCX 26 KB) [file 10238_2024_1438_MOESM10_ESM.docx]

**Supplementary Table 6.** Summary of studies investigating diagnostic accuracy of the neutrophil-to-lymphocyte ratio and the platelet-to-lymphocyte ratio for the presence of infections in patients with systemic lupus erythematosus.

| **Study** | **Study design** | **N** | **Age (years)** | **M/F** | **AUC (95% CI)**  **NLR**  **PLR** | **Cut-off**  **NLR**  **PLR** | **Sensitivity (%)**  **NLR**  **PLR** | **Specificity (%)**  **NLR**  **PLR** |
| --- | --- | --- | --- | --- | --- | --- | --- | --- |
| Kim HA et al. 2017, South Korea [33] | P | 120 | 33 | 9/111 | 0.872 (0.809-0.936)  NR | 5.7  NR | 0.75  NR | 0.9  NR |
| Broca-Garcia BE et al. 2019, Mexico [36] | P | 40 | 40 | 5/35 | 0.768 (0.626-0.927)  NR | 6.3  NR | 0.7  NR | 0.85  NR |
| Li Z et al. 2020, China [41] | P | 164 | 45 | 48/116 | 0.624 (0.525-0.723)  NR | 3.24  NR | 0.696  NR | 0.568  NR |
| Carvalho JS et al. 2022, Brazil [45] | R | 104 | 35 | 12/92 | 0.61 (0.502-0.708)  0.664 (0.554-0.773) | 3.5  151.4 | 0.65  0.71 | 0.58  0.53 |
| Mehta P et al. 2022, India [47] | P | 110 | 25 | 12/98 | NR  NR | NR  NR | 0.44  NR | 0.8  NR |
| Musunuri B et al. 2022, India [49] | P | 152 | 27 | 10/142 | 0.743 (NR)  0.668 (NR) | 4.19  422.6 | 0.71  0.676 | 0.732  0.689 |

Legend: NR, not reported; P, prospective; R, retrospective; M/F, male to female ratio; AUC, area under the curve; NLR, neutrophil-to-lymphocyte ratio; PLR, platelet-to-lymphocyte ratio.
